# Feature-based fuzzy connectedness segmentation of ultrasound images with an object completion step

**DOI:** 10.1016/j.media.2015.07.002

**Published:** 2015-12

**Authors:** Sylvia Rueda, Caroline L. Knight, Aris T. Papageorghiou, J. Alison Noble

**Affiliations:** a Centre of Excellence in Personalised Healthcare, Institute of Biomedical Engineering, Department of Engineering Science, University of Oxford, Old Road Campus Research Building, Headington, OX3 7DQ Oxford, UK; b Nuffield Department of Obstetrics & Gynaecology, University of Oxford, Oxford, U.K; c Oxford Maternal & Perinatal Health Institute, Green Templeton College, University of Oxford, Oxford, UK

**Keywords:** Image segmentation, Ultrasound, Shape completion, Fetal imaging, Image quality

## Abstract

Medical ultrasound (US) image segmentation and quantification can be challenging due to signal dropouts, missing boundaries, and presence of speckle, which gives images of similar objects quite different appearance. Typically, purely intensity-based methods do not lead to a good segmentation of the structures of interest. Prior work has shown that local phase and feature asymmetry, derived from the monogenic signal, extract structural information from US images. This paper proposes a new US segmentation approach based on the fuzzy connectedness framework. The approach uses local phase and feature asymmetry to define a novel affinity function, which drives the segmentation algorithm, incorporates a shape-based object completion step, and regularises the result by mean curvature flow. To appreciate the accuracy and robustness of the methodology across clinical data of varying appearance and quality, a novel entropy-based quantitative image quality assessment of the different regions of interest is introduced. The new method is applied to 81 US images of the fetal arm acquired at multiple gestational ages, as a means to define a new automated image-based biomarker of fetal nutrition. Quantitative and qualitative evaluation shows that the segmentation method is comparable to manual delineations and robust across image qualities that are typical of clinical practice.

## Abstract (author highlights)

**Highligts**
• Novel US segmentation approach based on the fuzzy connectedness framework.
• Use of local phase and feature asymmetry to define affinity function.
• Shape-based object completion step to detect and complete one or more gaps.
• Novel regional entropy-based quantitative image quality assessment approach.
• Method performs well across a variety of image qualities from clinical practice.

## Graphical abstract

**Figure:**
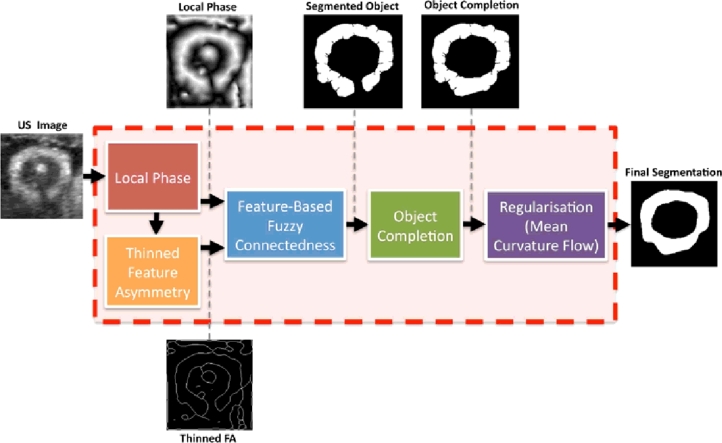

## 1 Introduction

Organ and tissue delineation is essential for underpinning image-based measurements of organ dimensions or tissue region properties. However, manual delineation is a tedious, subjective, time-consuming, and error prone task highly related to the image characteristics and the expertise of the observer. Development of automatic methods for quantitative analysis is especially challenging in ultrasound (US) images, where objects can show strong inhomogeneities and boundaries, can appear fuzzy or are not visible, and in the case of fetal analysis (which motivated this work) further issues are the change in appearance across gestational age and the challenge of fetal movement artefacts. Typically, purely intensity-based methods do not lead to good segmentation results. Several approaches are available at present for segmenting *B*-mode US images (Noble and Boukerroui, 2006). Among these, the use of local phase, derived from the monogenic signal (Felsberg and Sommer, 2001), has proven useful for a variety of image analysis tasks including segmentation (Belaid, Boukerroui, Maingourd, Lerallut, 2011, Hacihaliloglu, Abugharbieh, Hodgson, Rohling, 2008), registration (Mellor and Brady, 2005), image enhancement (Boukerroui et al., 2001), tissue characterization (Szilágyi et al., 2009), and feature detection (Bridge, Noble, 2015, Mulet-Parada, Noble, 2000, Rahmatullah, Papageorghiou, Noble, 2012), since local-phase methods extract structural image information while being invariant to contrast.

Among the many image segmentation methods that are currently available, the fuzzy connectedness (FC) framework can potentially deal with the fuzziness inherently present in US images and is defined by a discrete mathematical formulation, which makes it easy to implement. Fuzzy connectedness (Udupa, Saha, 2003, Udupa, Samarasekera, 1996) is a region-based approach. The main idea consists of defining the strength of local “hanging togetherness” of pixels within an image taking into account their spatial relationship and their intensity similarities within the object of interest. Some variants such as Iterative Relative Fuzzy Connectedness have been shown to be equivalent to other segmentation methods such as graph cuts (Ciesielski et al., 2012) and the Absolute Fuzzy Connectedness with gradient based affinity to level-sets (Ciesielski and Udupa, 2012). This approach has proven to be effective in terms of precision, accuracy, and efficiency (Udupa et al., 2006) in segmenting tissues in the presence of intensity gradation in MR and CT images over numerous applications (e.g. Multiple Sclerosis, Udupa et al., 2001, artery-vein separation, Lei et al., 2001, brain tumour segmentation, Moonis et al., 2002, etc.). To our knowledge this article is the first to consider design of a solution specially formulated for US images.

The particular segmentation challenge considered in this paper is 2D fetal US image segmentation. Previous automatic methods developed for this task have focused on extracting standard biometry (size) parameters over a narrow gestational age range. Examples include methods developed for the fetal head (Chalana, Winter, Cyr, Haynor, Kim, 1996, Hanna, Youssef, 1997, Lu, Tan, 2000, Lu, Tan, Floyd, 2005, Pathak, Chalana, Kim, 1997, Pathak, Chalana, Kim, 1997), the fetal femur (Rahmatullah, Besar, 2009, Shrimali, Anand, Kumar, 2009, Thomas, Jeanty, Peters, Parrish, 1991, Thomas, Peters, Jeanty, 1991), and the fetal abdomen (Chalana, Winter, Cyr, Haynor, Kim, 1996, Ciurte, Rueda, Bresson, Nedevschi, Papageorghiou, Noble, Bach-Cuadra, 2012, Nithya, Madheswaran, 2009, Yu, Wang, Chen, Shen, 2008) by using active contour models, morphological operators, machine learning, deformable models, or Hough transform approaches. Further, there are a limited number of papers in the literature that have proposed to estimate multiple standard fetal biometric measurements using a general method (Carneiro, Georgescu, Good, Comaniciu, 2008, Yu, Wang, Chen, 2008). The latter work, was subsequently translated into a commercial tool, called Auto OB (Carneiro et al., 2008b). Finally, state-of-the-art segmentation methods for automatic biometry of the fetal head and femur were recently compared on ultrasound data acquired across gestational age in a recent medical image analysis challenge (Rueda et al., 2014).

In 3D ultrasound, Yaqub et al. (2014a) has considered segmentation of 3D femur bone volumes using Random Forests, Cuingnet et al. (2013) has considered automatic detection and alignment of the fetal head from 3D US volumes, and automatic standard plane localization from 3D ultrasound volumes has been considered for the fetal abdomen (Ni et al., 2014) and 3D fetal neurosonography (Carneiro, Amat, Georgescu, Good, Comaniciu, 2008, Yaqub, Kopuri, Rueda, Sullivan, McCormick, Noble, 2014). Other fetal organs that have been investigated from a quantitative biomedical image analysis perspective are the fetal lungs (Prakash et al., 2002), heart (Deng, Wang, Shen, Chen, 2012, Dindoyal, Lambrou, Deng, Ruff, Linney, Rodeck, Todd-Pokropek, 2005, Veronese, Cosmi, Visentin, Poletti, Grisan, 2012), fetal face (Feng et al., 2009), and the fetal brain (Namburete, Noble, 2013, Namburete, Ramatullah, Noble, 2012, Namburete, Stebbing, Kemp, Yaqub, Papageorghiou, Noble, 2015, Gutiérrez Becker, Cosío, Huerta, Benavides-Serralde, 2010, Yaqub, Cuingnet, Napolitano, Roundhill, Papageorghiou, Ardon, Noble, 2013).

Most previous studies were designed to work over a particular gestational age range (particularly 18–22 weeks which corresponds to the interval of the abnormality screening scan). This avoids the main challenges (articulated later in the paper) of developing segmentation solutions applicable across gestation. To our knowledge, the only previous work to propose estimation of a fetal ultrasound biomarker across a large gestational age range is the framework (Namburete, Stebbing, Kemp, Yaqub, Papageorghiou, Noble, 2015, Namburete, Yaqub, Kemp, Papageorghiou, Noble, 2014) that accurately predicts the gestational age of the fetus based on analysis of brain structures using a regression forest model.

None of the previous works have attempted to relate the quality of the images to the quality of segmentation results which is an original contribution of this paper, and most prior work only uses a small number of images to develop and validate a method.

As with the work of Namburete et al. (2014) and Namburete et al. (2015) the development of this method was motivated by the clinical need for cost-effective and simple image-based biomarker tools for supporting pregnancy care in the developing world. Ultrasound-based tools are natural to consider for this purpose. Specifically, fetal adipose tissue in the limbs has been shown to be representative of fetal nutritional state (Larciprete et al., 2003), and its quantification has been hypothesised to be a good indicator of fetal growth (Bernstein et al., 1997). Motivated by this, recent clinical studies by our group (Knight, Rueda, Noble, Papageorghiou, 2012, Knight, Rueda, Noble, Papageorghiou, 2012) have shown that estimation of adipose tissue from US images of fetal limbs (fat and fat-free regions), via manual delineation, can characterise differences between healthy fetuses and neonates and relates to fetal nutrition. The method proposed in the paper was designed to automate estimation of this image-based biomarker. We are not aware of any previous work on automatic segmentation of arm adipose tissue on fetal US images.

The contributions of this article are three fold. First, we consider how to extend the Absolute Fuzzy Connectedness (AFC) approach to US images by defining a new affinity function. This is done by incorporating information extracted from local phase features instead of image intensities into the AFC framework affinity function. The resulting local phase-based FC framework becomes invariant to contrast and thus is well-suited for US image segmentation. Second, we present a new shape-based method for object completion of one or more ‘gaps’, to deal with missing information resulting from regions without an ultrasonic signal response (for example due to ultrasonic shadows). The result of object completion is then regularised by mean curvature flow. Thirdly, we introduce an approach to quantify the image quality (which can vary considerably between US image acquisitions) of an ultrasound image segmentation validation dataset to appreciate the accuracy and robustness of the developed analysis methodology across clinical data of varying appearance and representative of potential real world applications. The latter is especially important for US image analysis methods, where results are normally linked to the quality of the images and general practice (with few exceptions) is to report findings on good acoustic window data.

Preliminary versions of parts of this article appeared in Rueda et al. (2011); 2012b). The present paper presents a more general formulation of the complete analysis method, an in-depth evaluation on clinical data, and the new method for quantitative US image quality assessment is introduced for the first time.

The outline of the remainder of the paper is as follows. In Section 2, the overall segmentation framework is introduced and explained in detail. Qualitative and quantitative evaluations, including the proposed method of quantitative image quality assessment, are presented in Section 3. A discussion and conclusions are given in Section 4.

## 2 Segmentation framework

**Fig. 1:**
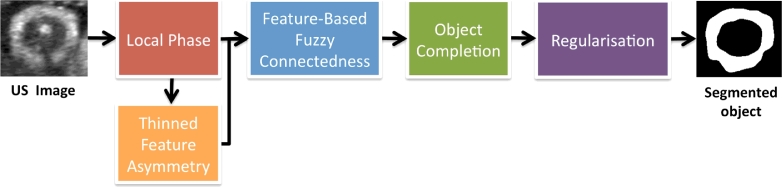
Proposed feature-based segmentation framework.

The overall segmentation framework is composed of several steps summarised in Fig. 1. Each step is explained in the following subsections.

### 2.1 Local phase derived from the monogenic signal

Let $f_{A}\left(t\right)$ be the complex analytic signal derived from *f*(*t*) and its Hilbert transform $f_{H}\left(t\right)$ as $f_{A}\left(t\right)=f\left(t\right)-if_{H}\left(t\right)$. This representation allows the extraction of the local amplitude (energy) *A*(*t*) and local phase φ(*t*) of *f*(*t*) defined as $A\left(t\right)=∥f_{A}\left(t\right)∥=\sqrt{f^{2}\left(t\right)+f_{H}^{2}\left(t\right)},$ and $\varphi \left(t\right)=arctan(f_{H(t)}/f\left(t\right)),$ respectively.

The monogenic signal (Felsberg and Sommer, 2001) *I_M_*(*x, y*) of an image *I*(*x, y*) generalises the analytic signal to 2D (and higher dimensions) using the Riesz transform instead of the Hilbert transform. From the monogenic signal, the local phase, local energy, and local orientation can be estimated.

In the spatial domain, the convolution kernels of the Riesz transform are defined as

(1) $$\begin{array}{c} h_{1}\left(x,y\right)=\frac{x}{2\pi {\left(x^{2}+y^{2}\right)}^{\frac{3}{2}}}\;\mathrm{and}\;h_{2}\left(x,y\right)=\frac{y}{2\pi {\left(x^{2}+y^{2}\right)}^{\frac{3}{2}}}, \end{array}$$

which in the frequency domain are expressed as

(2) $$\begin{array}{c} H_{1}\left(u,v\right)=\frac{u}{\sqrt{u^{2}+v^{2}}}\;\text{and}\;H_{2}\left(u,v\right)=\frac{v}{\sqrt{u^{2}+v^{2}}}, \end{array}$$

respectively. The quadrature pair (*H*_1_, *H*_2_) define the Riesz transform.

The implementation requires a pair of bandpass quadrature filters to extract the local properties of an image (amplitude, phase, and orientation). The image I(x), where x=(x,y), is first convolved with a bandpass filter b(x), to give $I_{b}\left(x\right)=b\left(x\right)⊗I\left(x\right),$ where ⊗ denotes the convolution operation. The bandpass filter chosen was a Gaussian derivative filter (Boukerroui et al., 2004) defined in the frequency domain as

(3) $$\begin{array}{c} B\left(u\right)=|u|\exp\left(-u^{2}\sigma^{2}\right), \end{array}$$

where u=(u,v) and *σ* is the selected scale of the filter.

**Fig. 2:**
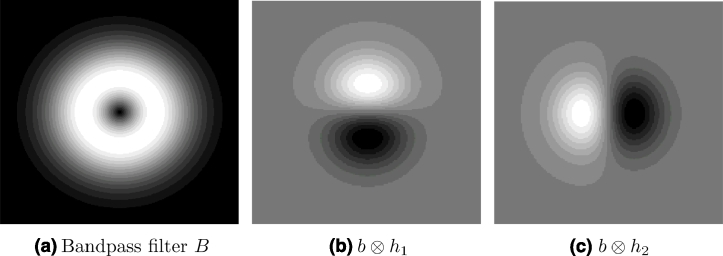
Gaussian derivative kernels for *σ* > 0. (a) Even component; (b–c) quadrature pair of odd filters.

This filter was empirically chosen, giving better visual maps than other candidate bandpass filters. This is not a critical part of the methodology and other filters, such as Cauchy (Boukerroui et al., 2004), may be better suited for other applications. An example of a Gaussian derivative bandpass filter and the resulting bandpass quadrature pair of odd filters is shown in Fig. 2.

The monogenic signal $I_{M}\left(x\right)$ of I(x) is then expressed as

(4) $$\begin{array}{c} I_{M}\left(x\right)=\left(I_{b}\left(x\right),h_{1}\left(x\right)⊗I_{b}\left(x\right),h_{2}\left(x\right)⊗I_{b}\left(x\right)\right). \end{array}$$

The *local amplitude* (energy) A(x), *local phase*φ(x), and *local orientation*θ(x) of I(x) are derived from $I_{M}\left(x\right)$ and defined as

(5) $$\begin{array}{c} A\left(x\right)=\sqrt{I_{b}{\left(x\right)}^{2}+{\left(h_{1}\left(x\right)⊗I_{b}\left(x\right)\right)}^{2}+{\left(h_{2}\left(x\right)⊗I_{b}\left(x\right)\right)}^{2}}, \end{array}$$

(6) $$\begin{array}{c} \varphi \left(x\right)=\arctan\left(\frac{I_{b}\left(x\right)}{\sqrt{{\left(h_{1}\left(x\right)⊗I_{b}\left(x\right)\right)}^{2}+{\left(h_{2}\left(x\right)⊗I_{b}\left(x\right)\right)}^{2}}}\right), \end{array}$$

(7) $$\begin{array}{c} \text{and}\;\theta \left(x\right)=\arctan\left(\frac{h_{2}\left(x\right)⊗I_{b}\left(x\right)}{h_{1}\left(x\right)⊗I_{b}\left(x\right)}\right), \end{array}$$

respectively. The structural information is invariant to contrast and contained in the local phase, whereas the local amplitude represents the energy, which is dependent on intensity values. An example of local phase image can be seen in Fig. 15(a) for the image in Fig. 11(b).

### 2.2 Feature asymmetry

Computing the local phase at different scales, allows one to detect step edge features as points where there is local phase congruency (Kovesi, 1999). In other words, a positive step edge will have a local phase value of 0° and a negative step edge will have a value of 180°. To detect step edge features, we use the *feature asymmetry* FA measure, calculated over a number of scales, and defined as

(8) $$\begin{array}{c} \mathrm{FA}\left(x\right)=\frac{1}{N}\sum_{s}\frac{\left\lfloor |da\mathrm{odd}{\left(x\right)}_{s}|-|\text{even}{\left(x\right)}_{s}|-T_{s}\right\rfloor }{\sqrt{\text{even}{\left(x\right)}_{s}^{2}+da\mathrm{odd}{\left(x\right)}_{s}^{2}}+ɛ}, \end{array}$$

where $\text{even}\left(x\right)=I_{b}\left(x\right),$$da\mathrm{odd}\left(x\right)=(h_{1}\left(x\right)⊗I_{b}\left(x\right),$$h_{2}\left(x\right)⊗I_{b}\left(x\right)),$ ⌊.⌋ sets to zero the negative values, *s* represents the scale, *N* is the total number of scales, ε is a constant that avoids the division by zero (typically ɛ=0.01), and *T_s_* is an orientation independent threshold that controls the spurious responses to noise at scale *s* (Kovesi, 1999, Mulet-Parada, Noble, 2000). *T_s_* can be estimated from statistical properties of the energy response (Kovesi, 1999) or by approximating the statistical mode (Mulet-Parada and Noble, 2000).

The FA image consists of thick detected edges with values close to 1 and with homogeneous regions close to 0 values. An example of a feature asymmetry image can be seen in Fig. 15(b) for the image in Fig. 11(b). However, a good localization of the edges of the object of interest is essential in this framework. Therefore, we need a technique to thin the feature asymmetry edge features while retaining most of the information present in the FA image. A *non-maximal suppression* technique (such as Sonka et al., 2008) could be used for this, but it will be unable to retain information in directions other than the local orientation direction at each edge pixel. Therefore, a *modified non-maximal suppression* technique was developed. First, for each pixel in the FA image, non-maximal suppression is performed in all possible directions. Then, at each pixel, the maximum value among all directions is retained. This strategy captures the relevant edge information with good localization while retaining the same intensity present in the FA image, thus obtaining the edge map *E* that will be used in this work. An example can be seen in Fig. 15(c) for the feature asymmetry image in Fig. 15(b).

### 2.3 Feature-based fuzzy connectedness

Although several variations of the fuzzy connectedness method exist (e.g. Iterative Relative Fuzzy Connectedness - IRFC, Relative Fuzzy Connectedness - RFC), in this paper we have chosen to employ one of the original formulations of FC, namely Absolute Fuzzy Connectedness (AFC), to study how it would perform using the affinities specially formulated for US imagery.

The Absolute Fuzzy Connectedness strategy (Udupa, Saha, 2003, Udupa, Samarasekera, 1996) is based on a *global* fuzzy relation that assigns a strength of connectedness to every pair of pixels in an image to define objects via dynamic programming. The key step of this region-based approach relies on the definition of a *local* fuzzy relation *μ_κ_*, called *affinity*, which defines the local “hanging togetherness” between any two adjacent pixels. If two pixels *c* and *d* are adjacent, the affinity depends on how homogeneous the region is and on how close the intensity values at *c* and *d* are from the expected intensity value of the object of interest. The affinity is equal to 0 for non-adjacent pixels.

The affinity values are used to define a *global* relation, called Fuzzy Connectedness, where the strength of connectedness between any two pixels is calculated as the largest of the strengths of all paths between *c* and *d* on the discrete image grid. Each path corresponds to a sequence of adjacent pixels starting from *c* and finishing in *d* and has a corresponding strength value, which is the smallest affinity of any pair of consecutive pixels along the path (weakest link). The Absolute Fuzzy Connectedness is represented as a connectivity map, where the object of interest is obtained by thresholding the image at *T_FC_*. The detailed mathematical description of the method can be found in (Udupa, Saha, 2003, Udupa, Samarasekera, 1996).

The initialisation of the general method is based on manually placing one or several seeds within the object of interest. A minimal training stage is required once to define the typical mean and standard deviation of the intensity values of the object of interest.

The AFC framework was adapted to US segmentation by defining a new affinity function that uses structural and edge feature information instead of intensities and intensity gradients. The affinity function was designed as follows. Assume that every fuzzy subset A in a set is characterised by its membership function $\mu_{A}$ with values in [0, 1]. Given an image, the affinity is composed of three factors: an adjacency component *μ_α_*, an object feature-based component *μ_ϕ_*, and a homogeneity-based component *μ_ψ_*. The adjacency component *μ_α_* is a non-increasing function of the distance in pixels (i.e. integers) ∥c−d∥ defined as

(9) $$\begin{array}{c} \mu_{\alpha }\left(c,d\right)=\left\{ \begin{array}{cc} 1, & \;\text{if}\;c=d\;\text{or}\;∥c-d∥=1,\\ 0, & \;\text{otherwise.} \end{array}\right. \end{array}$$

In the original framework by Udupa and Samarasekera (1996), the object feature-based component $\mu_{\phi_{1}}$ was defined based on the intensities of the image, whereas the homogeneity-based component $\mu_{\psi_{1}}$ was a measure of intensity gradient. The proposed method incorporates the local phase information into the object-feature based component, extracting structural information and making the image invariant to contrast. The edge map *E*, derived from the feature asymmetry image, directly gives a measure of homogeneity, since smooth regions have small values and regions near boundaries have large values (cf. Section 2.2). Therefore, it is natural to consider it in the definition of the homogeneity-based component. Let φ(*c*) be the local phase at pixel *c* and *E*(*c, d*) the thinned pixel edge derived from feature asymmetry between pixels *c* and *d*. The homogeneity-based component $\mu_{\psi_{2}}$ will have a high affinity in homogeneous regions and small affinity at the edges. Since *E* is close to 0 in homogeneous regions and close to 1 at edge features, we can express the homogeneity component as

(10) $$\begin{array}{c} \mu_{\psi_{2}}\left(c,d\right)=1-E\left(c,d\right)=g_{3}\left(E\left(c,d\right)\right), \end{array}$$

where *g*_3_ is a function of *E*(*c, d*). The object feature-based component $\mu_{\phi_{2}}$ takes into account characteristic features of the object of interest. In this paper, a recent formulation (Ciesielski and Udupa, 2010) was applied directly to the local phase image instead of intensities, as follows:

(11) $$\begin{array}{c} \mu_{\phi_{2}}\left(c,d\right)=e^{-\max{\left\{∥\varphi \left(c\right)-m_{o}∥,∥\varphi \left(d\right)-m_{o}∥\right\}}^{2}/2\sigma_{o}^{2}}=g_{4}\left(\varphi \left(c\right),\varphi \left(d\right)\right), \end{array}$$

where *m_o_* and *σ_o_* are the mean and standard deviation of the intensity values of the object of interest, previously calculated in a training stage, and *g*_4_ is a function of φ(*c*) and φ(*d*).

There exist several ways of combining the affinity components to form the fuzzy affinity *μ_κ_* (Ciesielski and Udupa, 2010). One general form commonly used is

(12) $$\begin{array}{c} \mu_{\kappa }\left(c,d\right)=\mu_{\alpha }\left(c,d\right)\left[\omega_{1}g_{1}\left(I\left(c\right),I\left(d\right)\right)+\omega_{2}g_{2}\left(I\left(c\right),I\left(d\right)\right)\right], \end{array}$$

where *I*(*c*) and *I*(*d*) correspond to the intensities at pixels *c* and *d*, respectively (Udupa and Samarasekera, 1996), $g_{1}\left(I\left(c\right),I\left(d\right)\right)=\mu_{\psi_{1}}\left(c,d\right),$ and $g_{2}\left(I\left(c\right),I\left(d\right)\right)=\mu_{\phi_{1}}\left(c,d\right)$. The equivalent affinity function $\mu_{\kappa }^{*}$ for the proposed approach is expressed as

(13) $$\begin{array}{c} \mu_{\kappa }^{*}\left(c,d\right)=\mu_{\alpha }\left(c,d\right)\left[\omega_{1}g_{3}\left(E\left(c,d\right)\right)+\omega_{2}g_{4}\left(\varphi \left(c\right),\varphi \left(d\right)\right)\right], \end{array}$$

where $\omega_{1}+\omega_{2}=1,$ and with *g*_3_ and *g*_4_ as defined in (10) and (11), respectively.

### 2.4 Delineating closed regions

The segmentation resulting from the feature-based FC only incorporates regions of the object of interest present in the image. However, it is unable to delineate object boundaries in shadowed areas (e.g. shadow under the humerus bone in Fig. 11(b)), as there is no ultrasonic signal response from these regions. Furthermore, in some cases, the object of interest can be formed by several connected pieces with missing information between them that we would like to retrieve. To overcome this, a new object completion technique has been developed. This first detects the region of the object of interest with missing information, and then fills the gap(s) in by using local shape constraints (Rueda et al., 2008). In the preliminary version of the method (Rueda et al., 2012b), only one gap was corrected. In this paper we have generalised the approach to the detection and completion of any number of gaps appearing in the object of interest after segmentation. The object completion step is described in the next subsection.

#### 2.4.1 *c*-scale shape descriptor

**Fig. 3:**
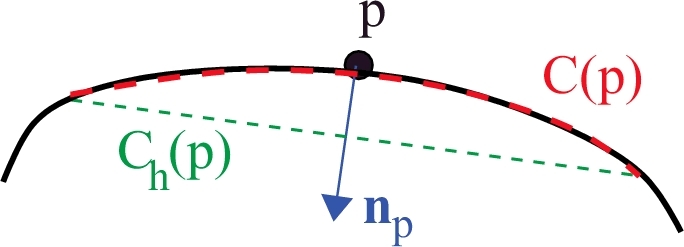
*c*-scale estimation at *p* in a piece of boundary (black curve). *C*(*p*) is the *c*-scale segment associated with *p* and *C_h_*(*p*) is the *c*-scale value corresponding to *C*(*p*). (For interpretation of the references to colour in this figure legend, the reader is referred to the web version of this article).

At each point *p* on a boundary, a local curvature scale segment (Rueda et al., 2008), called *c-scale segment C*(*p*), is defined as the set of connected points at a distance smaller than a threshold *t* from the line connecting the two end points of the set (red dashed curve in Fig. 3). Each *C*(*p*) is obtained after symmetrically and progressively examining the adjacent boundary elements to *p* until the distance is no greater than a threshold *t*.

A *c-scale value C_h_*(*p*) (green dashed line in Fig. 3) can then be obtained as the chord length corresponding to *C*(*p*), which is the length of the straight-line segment between the end points in *C*(*p*). Large *C_h_*(*p*) values indicate small curvature at *p*, whereas small values denote high curvature (Rueda et al., 2008). Values of *c*-scale are very useful in estimating actual segments and their curvature by considering the local morphometric scale of the object, and are independent of digital effects and noise. The *c*-scale method has proven to be robust in obtaining a complete description of shape directly applied to digital boundaries. More details can be found in Rueda et al. (2008).

An extension of this implementation was developed to obtain the normal $n_{p}$ at each point *p* in the boundary as the line perpendicular to *C_h_*(*p*) passing through *p* (Fig. 3). The direction of the normals is always selected pointing to the inside of the object. Only the normal information is needed for the object completion step, which is described in the following.

#### 2.4.2 Object completion

The object completion is performed in three steps: convex hull boundary extraction, gap detection, and gap completion.

**Fig. 4:**
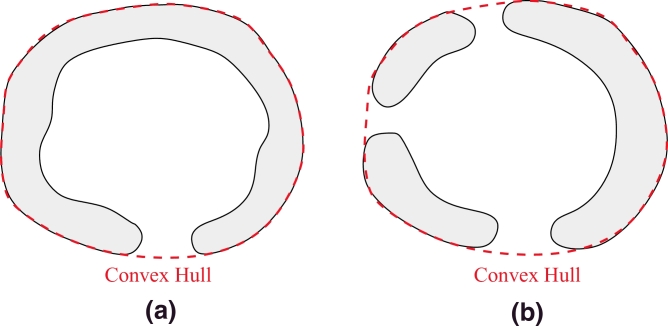
Convex hull boundary for (a) one connected component; and (b) several connected components. The convex hull boundary is represented by a red dashed line. (For interpretation of the references to colour in this figure legend, the reader is referred to the web version of this article).

First, the convex hull of the segmentation result is computed and its boundary extracted (Fig. 4). If the segmented object is composed by several connected components, the convex hull contains all of them, as shown in Fig. 4(b). In the following, the completion strategy is illustrated on an example with three gaps to describe a general case. In our application, all the objects will have at least one gap to complete.

**Fig. 5:**
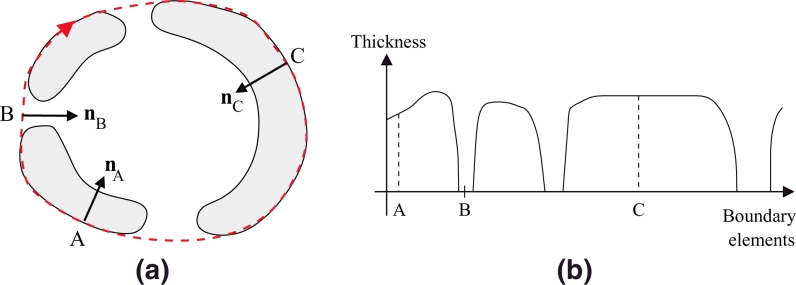
Gap detection. (a) Normals are calculated around the convex hull boundary using the *c*-scale shape descriptor. (b) Thickness of the segmented object is calculated at each boundary element of the convex hull. Zero thickness indicates the presence of a gap.

**Fig. 6:**
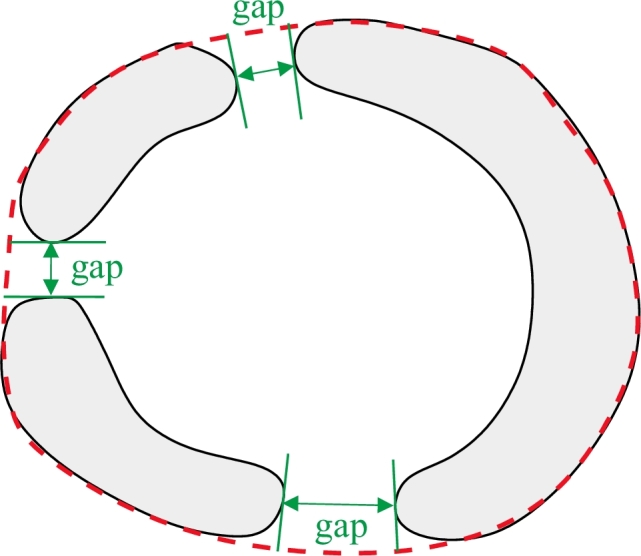
Gaps detected after identification of zero thickness regions as shown in Fig. 5.

For each boundary element in the convex hull boundary, the *c*-scale shape descriptor (Rueda et al., 2008) is used to define the tangent (chord) at each point in the curve. From the tangents, the normals to the convex hull boundary are calculated at each boundary point in the direction pointing towards the inside of the object (Fig. 5(a)). Then, the binary intersection between each normal and the segmented object (resulting from the feature-based fuzzy connectedness step) is retrieved and the connected object closest to the convex hull boundary element is retained. The width (thickness) is then calculated (Fig. 5(b)) by measuring the length of the connected object previously extracted for each boundary element of the convex hull. The gap(s) is(are) detected by finding the region(s) with zero width, as represented in Fig. 6.

**Fig. 7:**
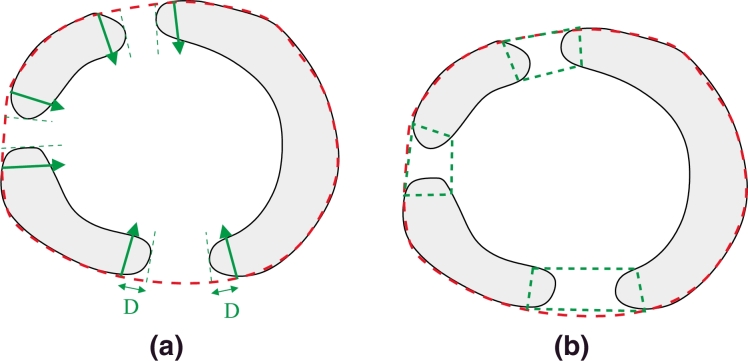
Gap completion using polygons. (a) Normals are found at either side of the gap at a fixed distance *D*. (b) A polygon is constructed using the normals and the segmented objects. The polygons are represented by green dashed lines. (For interpretation of the references to colour in this figure legend, the reader is referred to the web version of this article).

The last step consists of filling in the gap(s) in the segmented object. Two normals on each side of each detected gap are then identified at a fixed distance *D* (Fig. 7(a)). A polygon is constructed by connecting the two detected normals on each side of the hole at the level of the convex hull boundary from one side, and the segmented object boundary from the other side (Fig. 7(b)). The corrected object is obtained from the binary union between the polygon and the segmented object. Note that a different completion strategy could have been used instead of a polygon. For example, curvature information could be used to complete the gaps after detection using curves. However, in this case, we tried to follow the same strategy clinicians were using to complete the shapes in our particular application. Algorithm 1 summarises the object completion step.

### 2.5 Regularisation

The resulting object boundary is finally smoothed using a mean curvature flow (MCF)^1^ regularisation strategy (Sethian, 1999). The method is based on the evolution of the curve using implicit functions. The points in the contour are moved in the normal direction with a speed proportional to the curvature at each point. A Matlab toolbox (Mitchell, 2008) was used for this purpose.

### 2.6 Implementation details

Local phase and feature asymmetry were estimated as described in Sections 2.1 and 2.2. The bandpass filter used within this framework is a Gaussian derivative filter, as defined in Eq. (3). Since the scale considered for local phase calculation (Eq. 6) depends on the size of the structure of interest, two different scales were considered, one for gestational ages below 30 weeks (s=27), and one for gestational ages above or equal to 30 weeks (s=35). For the calculation of the feature asymmetry (Eq. 8), three scales were considered (N=3), with s=[23,25,27] for gestational ages below 30 weeks and s=[27,30,35] for gestational ages above 30 weeks. *T_s_* was obtained from statistical properties of the local phase image (Kovesi, 1999), and set to $T_{s}=0.155$.

Within the AFC framework (cf. Section 2.3), for the object feature-based component of affinity (11), the mean $m_{o}=2.44$ and the standard deviation $\sigma_{o}=3\times 0.086$ were estimated from a region of fat in the local phase image from a training stage performed on three images, since the local phase value of a region of fat was very similar among images and didn’t require a larger training set. The images used for training were not part of the evaluation set. The final affinity (13) was calculated with $\omega_{1}=\omega_{2}=0.5$. The method is multi-seeded with one or more seeds in the fat layer of the image used for initialisation. In the set of images used, most of the images required one seed and a few required more, if presenting disconnected fat appearance. No more than 5 seeds were used in any of the images. The object of interest was thresholded from the connectivity map by using $T_{FC}=0.85$. This value was set empirically but could have been automatised for each seed as in (Miranda et al., 2008).

The *c*-scale shape descriptor used for delineating closed regions of adipose tissue (cf. Section 2.4) only required one parameter *t*, which was set to t=5.

The AFC part of the method was implemented in Matlab, using C mex files for faster computation. The other steps of the presented framework were implemented in Matlab.

## 3 Results and evaluation

This section presents results of evaluation of the new segmentation method on a large clinical dataset. We begin by presenting the clinical image protocol in Section 3.1. The proposed framework is then directly compared to the original Absolute Fuzzy Connectedness method based on intensities and qualitatively and quantitatively against manual segmentations in Sections 3.2 and 3.3, respectively. We then, in Section 3.4, look more deeply at the performance of the algorithm by firstly characterizing the variability of the data in the clinical dataset, and use this characterization to gain better understand into the potential performance of the new segmentation method on real world clinical data.

### 3.1 Image acquisition

**Fig. 8:**
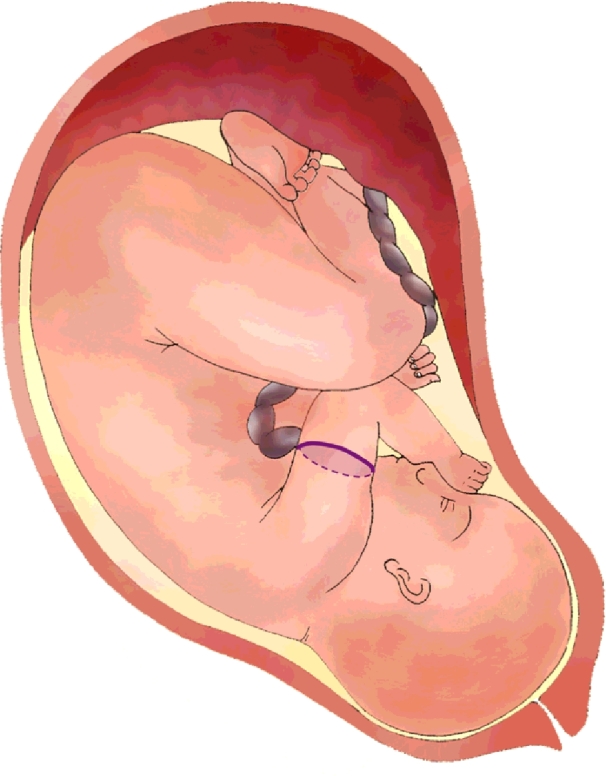
US cross-section acquisition of the fetal arm at mid-humeral level (purple cross-section).

**Fig. 9:**
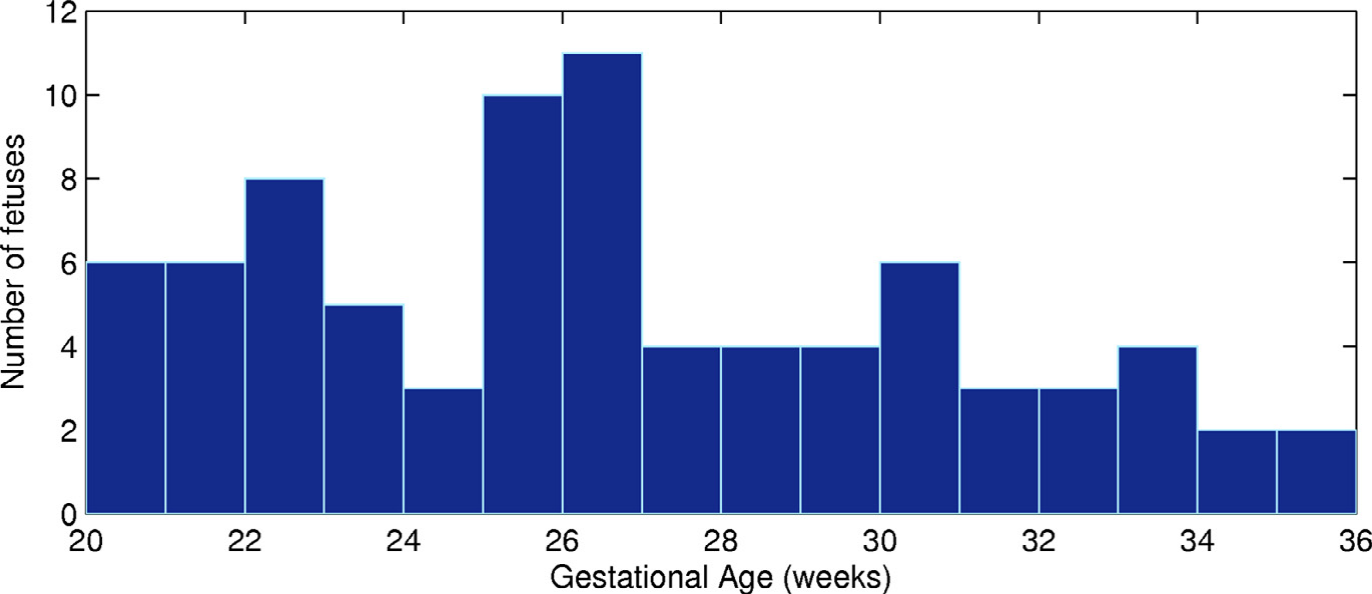
Distribution of gestational ages within the dataset.

The clinical dataset used for evaluation is 81 cross-sectional US images of the fetal arm across gestation acquired perpendicularly to the arm at mid-humeral level (Fig. 8) from 73 healthy fetuses between 20 and 36 weeks of gestation. For eight of these fetuses, images acquired at two different gestational ages were included. The distribution of gestational ages within the dataset is shown in Fig. 9.

The images were acquired with a Philips HD9 machine (Philips Ultrasound, Bothell, WA, USA) at the Nuffield Department of Obstetrics and Gynaecology, John Radcliffe Hospital, University of Oxford, Oxford, U.K. The fetuses involved in this clinical study are part of the INTERGROWTH-21st (2009)^2^ and INTERBIO-21st (2012)^3^ cohorts.

**Fig. 10:**
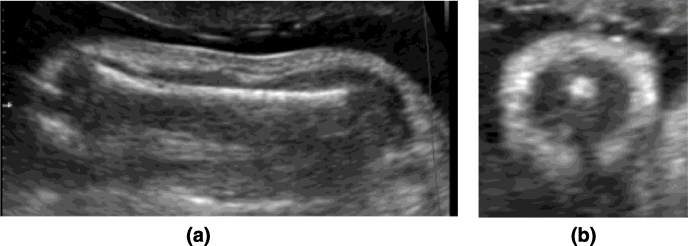
Fetal arm at 28 weeks of gestation. (a) Sagittal view with horizontal humerus bone. (b) Axial cross-section.

The protocol used for the acquisition of the US fetal arm cross-sections was as follows. First, the sagittal view of the humerus (Fig. 10(a)) was acquired to visualise the full humerus length longitudinally, ideally horizontal and in the centre of the screen. The probe was subsequently rotated 90° to obtain an axial cross-section of the arm at mid-humeral level (Fig. 10(b)).

**Fig. 11:**
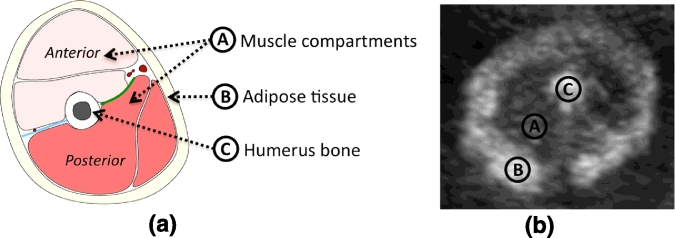
(a) Schematic of arm composition. (b) Arm cross-section of a 27 weeks fetus with characteristic shadow under the humerus bone, due to lack of ultrasonic signal response from that region. Notice intensity inhomogeneities within the adipose tissue (B).

Referring to Fig. 11, arm cross-sections are formed by a central hyperechoic bone surrounded by hypoechoic muscle and then an echodense fat layer. To ensure that the cross-sections were acquired perpendicular to the humerus, the probe was swept along the longitudinal axis of the humerus bone. If the axial view appeared to be perpendicular to the longitudinal axis then the image of the bone remained in the centre of the screen as the probe was moved. Adjustments were made until this was achieved and then returned to midpoint of humerus to acquire the 2D image.

**Fig. 12:**
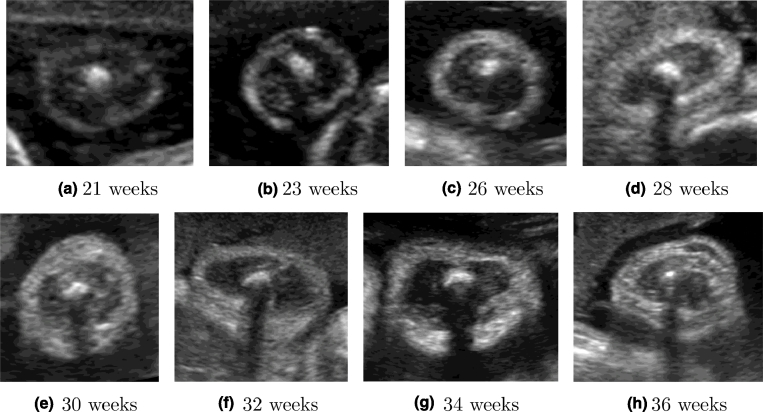
Image appearance of the fetal arm US cross-sections across gestational age.

**Fig. 13:**
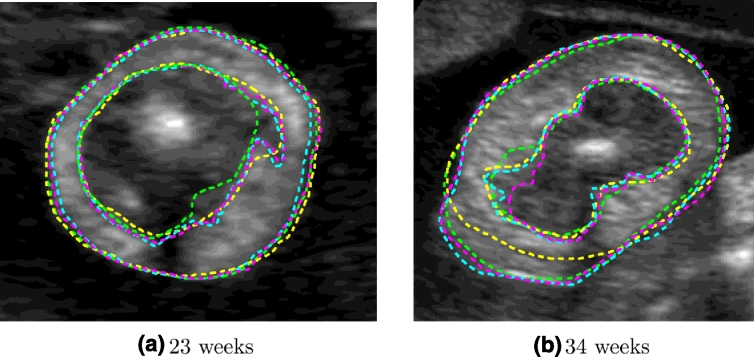
Manual segmentations performed twice by two different experts. (Expert 1: green and yellow contours. Expert 2: magenta and cyan contours). (For interpretation of the references to colour in this figure legend, the reader is referred to the web version of this article).

Image appearance was found to vary across gestation as illustrated in Fig. 12. The following general observations can be made to illustrate some of the challenges in image segmentation for this particular application. First, the shape of the fetal arm is not always circular and can vary globally or regionally due to the pressures created by surrounding structures - this is especially the case at later gestational ages. Second, the adipose tissue layer can produce pronounced intensity inhomogeneities, which are characteristic of this imaging modality. Changes in tissue texture can also create different speckle patterns at different gestational ages. Third, maternal and fetal tissues (e.g. as shown in Figs. 12(d, f and h) normally surround the arm, and can make the segmentation task difficult. Fourth, at early gestation, the layer of fat is very thin and hardly visible, since it is seen more clearly from 18–20 weeks. Fifth, the adipose tissue boundaries are usually fuzzy, which makes manual segmentation difficult and can cause discrepancies, as shown in Fig. 13, where the adipose tissue layers were manually segmented by two experts twice. Finally, observe that there is always a characteristic shadow appearing under the humerus bone (Figs. 11(b) and 12), which prevents the visualization of adipose tissue in that area. In manual segmentations, this region is typically approximated by joining the delineations on either side of the shadow by a straight line (Fig. 13).

### 3.2 Qualitative evaluation

**Fig. 14:**
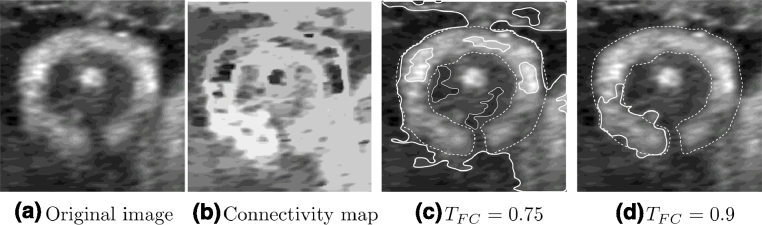
Intensity-based FC segmentation results. (a) Arm cross-section of a 28 weeks fetus. (b) Intensity-based FC connectivity map. (c) Segmentation for $T_{FC}=0.75$. (d) Segmentation for $T_{FC}=0.9$. Dashed lines: averaged manual segmentation. Continuous lines: FC segmentation results.

In this subsection, the proposed method is compared to the intensity-based Absolute Fuzzy Connectedness approach. However, due to the large intensity variability within the adipose tissue layer across the different images in our dataset, it proved impossible to set representative algorithm parameters for the intensity-based AFC method during the training stage. This situation is avoided when using local phase, as it is contrast invariant. A typical example of intensity-based segmentation is shown in Fig. 14. We observed that the intensity-based approach could not cope with the inhomogeneities present within the object of interest. This can be seen in Fig. 14(c), where high intensity regions within the adipose tissue area are not segmented. In this case, the variability of intensities within the region of interest is too high for the intensity-based method to correctly segment the overall adipose tissue layer.

**Fig. 15:**
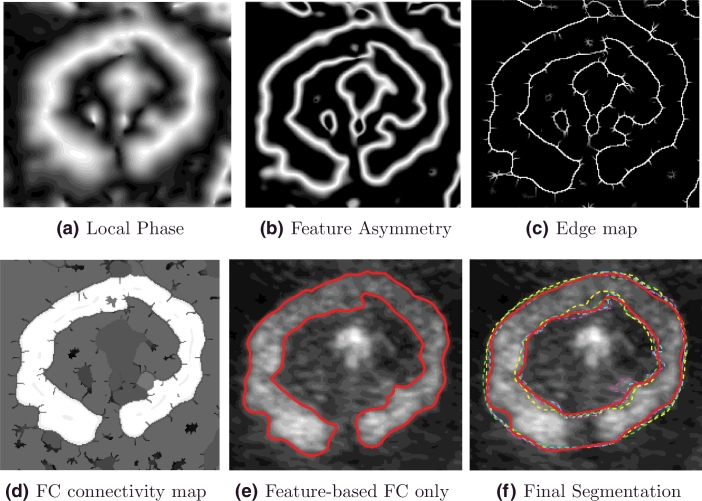
Main steps of the proposed method applied to Fig. 11(b). (a) Local Phase at σ=27. (b) Feature asymmetry with σ=23,σ=25,σ=27. (c) Edge map extracted from feature asymmetry using non-maximal suppression in all directions. (d) FC connectivity map. (e) Feature-based FC segmentation result. (f) Final segmentation obtained from feature-based FC after completion and regularisation. Manual segmentations are displayed in dashed lines. Continuous lines show the proposed segmentation results.

**Fig. 16:**
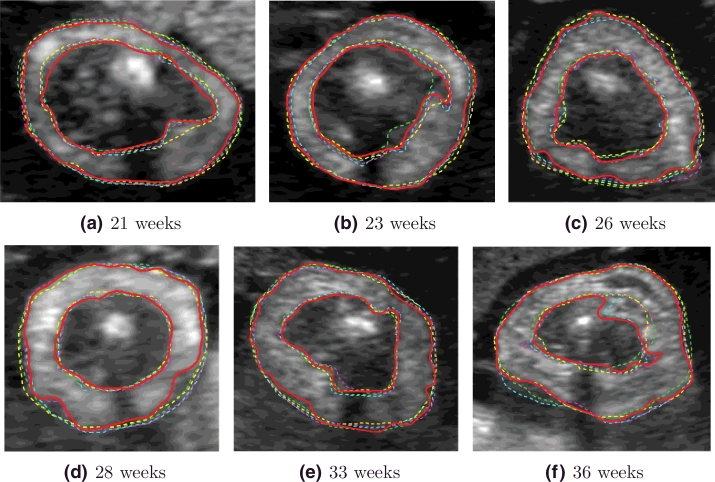
Segmentation results across gestational ages. Manual segmentations are displayed in dashed lines. (Expert 1: green and yellow lines. Expert 2: magenta and cyan lines.) Continuous red lines show the proposed segmentation results. (For interpretation of the references to colour in this figure legend, the reader is referred to the web version of this article).

Fig. 15 shows the outputs of key image analysis steps of the proposed methodology reported in this paper. Qualitative results comparing the automated method output with manual delineations at a number of discrete gestational ages are shown in Fig. 16. These results illustrate that the automated method appear visually similar to the manual delineations.

### 3.3 Quantitative evaluation

In this subsection, we quantitatively assess the proposed segmentation method by using a number of established region-based and distance-based metrics. First, region-based evaluation metrics, defined as area overlap measures, were selected as a way of assessing image segmentation precision (repeatability of the method) and accuracy (sensitivity and specificity). These metrics are as defined in Udupa et al. (2006). Experimental results were performed twice on each image of the dataset to assess the precision of the proposed method. Accuracy was reported as in Udupa et al. (2006), where delineation *sensitivity* is given by the true positive area fraction (TPAF) and delineation *specificity* by 1-FPAF where FPAF is the false positive area fraction. These two independent metrics are sufficient to quantify the general accuracy of a segmentation method. In each case, a larger value indicates a better segmentation performance.

We also report the commonly used Dice similarity metric. Distance-based metrics (maximum symmetric contour distance: MSD; average symmetric contour distance: ASD; and root mean square symmetric contour distance: RMSD), as described in Heimann et al. (2009), are also reported. As we do not have a “ground-truth” segmentation (the true arm composition is not known but only imaged indirectly), segmentation results were compared to manual delineations of the structures, segmented twice by each of the two experts. The results per image were averaged to obtain the overall performance for a particular expert and for all experts. More details on these particular metrics can be found in Rueda et al. (2014).

Table 1 presents the results for the intra and inter-observer variability assessment, obtained from the manually segmented images by two experts, segmented twice. The results show similar performance between experts, Expert 2 having slightly better results.

The segmentation evaluation results of the proposed approach were then compared to both experts and to the average manual segmentation, as shown in Table 2.

The proposed method performs similarly to manual delineation with mean results very close to those obtained for each metric calculated for the inter-expert variability (cf. Table 1) in terms of mean and standard deviation. The precision of the proposed segmentation approach, in terms of repeatability, was evaluated by repeating each segmentation twice using different seed locations as initialisation. The presented framework has a precision of 99.89%, which means that the results are very consistent. Very slight differences were noted in certain cases due to the selection of the seed positions. The repeatability is much higher than the one obtained from manual adipose tissue delineations (cf. Table 1) as expected.

### 3.4 Quantitative image quality assessment

This subsection firstly explains how we define image segmentation quality for our dataset and then interprets the automated algorithm performance with respect to the resulting image segmentation quality metrics.

It would be greatly beneficial to report segmentation results with a measure of image quality to characterise the dataset used and how well the method performs considering the quality of the images. However, establishing overall image quality measures is difficult, since the quality of images relies on tissue appearance. Ultrasound image quality can vary considerably between acquisitions, which may affect the performance of different segmentation methods.

In this paper, we propose a new solution to quantify image quality of a clinical dataset designed to provide deeper insight into segmentation performance. This is, to our knowledge, the first attempt to correlate segmentation results with a quantitative measure of US image quality.

US image quality depends on a number of factors including: the US machine (transducer, time-gain control, use of harmonics versus fundamental, persistence, and depth), the object being scanned (tissue properties (speckle), effects of attenuation (depth), shadows, and reverberations), and the orientation of the probe with respect to the object.

In fetal ultrasound imaging, object appearance varies with gestational age with the structures surrounding the object of interest showing high variability. Overall fetal US image quality tends to decrease towards later gestation as a result of the fetus becoming bigger with relatively less amniotic fluid, thus the fetal structures are more likely to be compressed resulting in the clear soft tissue/fluid interface. The bone density in the fetus also increases, creating more shadows and artefacts in the images. Another factor that can affect ultrasound image quality is the increase of maternal body mass index, attenuating the signal especially towards the end of pregnancy. Specifically, the proposed quantitative image quality assessment method relies on the principle that different tissues have specific sound propagation properties characterised by the complexity of the speckle pattern. These tissues do not evolve in the same way across gestation and surrounding structures vary depending on the acquisition angle and fetal position at that particular time. This is why an overall global image measure would not be as appropriate as a regional quality measure.

Traditional image processing quality measures such as SNR (signal-to-noise ratio) and CNR (contrast-to-noise ratio) rely on the estimation of the signal and noise from the entire image or regions. In the case of ultrasound image processing, and particularly the literature on speckle-reduction, speckle has typically been treated as the noise component. In our case a speckle-based measure would only capture texture differences, not contrast changes, and requires access to the RF signal to estimate a statistical model, for instance see Destrempes and Cloutier (2010); Raju and Srinivasan (2002). Further, the estimation of such models is non-trivial with accuracy depending on the block size used for parameter estimation for instance (Larrue and Noble, 2014). In our case, which is very typical of most image analysis work conducted with clinical groups, we have access to DICOM B-mode images only. Furthermore, due to the stochastic nature of the speckle patterns, using CNR directly for characterising echogeneity is sub-optimal, because the tissue contrast resolution depends on speckle variance and size. As these general image quality measures did not satisfy our needs we developed the approach described next.

The proposed new method quantifies the complexity of each region (resulting from the speckle distribution) and the relationship amongst tissues in an image without estimating a speckle model (statistical distribution). Specifically, first a manual image partitioning is made to each image resulting in manual delineations of different regions of interest. An entropy-based measure is then computed on each of the image partitions to estimate the information content in each region of interest. This quality measure is based on the appearance and complexity of each region and not on contrast, absolute intensity, or edge information. The probability density function of a region, denoted *p_r_*, is first estimated from the gray-level histogram of that region. The normalised histogram of a region *A_r_* is defined for each intensity value *a_k_* with k=1,…,M, *M* being the maximum number of intensity levels in *A_r_*. In our case, M=256. The entropy *H* of the random variable *A_r_* can then be calculated as

(14) $$\begin{array}{c} H\left(A_{r}\right)=-\sum_{k=1}^{M}p_{r}\left(a_{k}\right)\log_{2}p_{r}\left(a_{k}\right). \end{array}$$

The entropy difference between adjacent regions can then be calculated to assess the overall image quality (as a whole complexity measure) and correlated with the segmentation results.

**Fig. 17:**
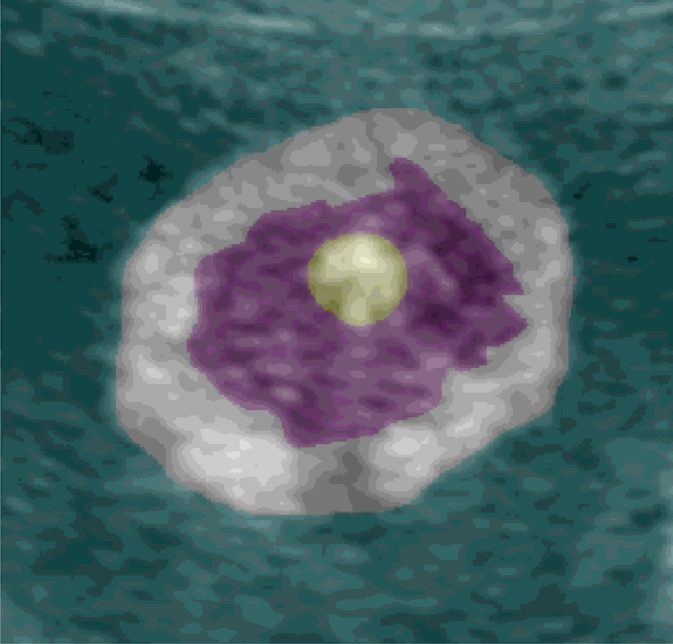
Fetal arm image partitioning into 4 regions (cyan: background region, white: adipose tissue layer, magenta: muscle region, and yellow: bone region) for a 27 week fetus. (For interpretation of the references to colour in this figure legend, the reader is referred to the web version of this article).

The proposed quantitative image quality assessment method was applied to the fetal arm dataset introduced in Section 3.1. The first step consisting of partitioning the images into different areas, is shown in Fig. 17. These regions were manually delineated in all the images of the dataset and the entropy calculated for each of these regions separately. The ideal image appearance, from an automated segmentation algorithm perspective, occur when the background and muscle regions have a hypoechoic appearance (dark) and the adipose tissue layer a hyperechoic appearance (bright), which should be clearly distinguishable from the surrounding tissues.

In the evaluation dataset used within this study, we deliberately (and unusually) selected examples with a wide range of image quality. The examples discussed below are typical examples taken from the whole dataset and were chosen to facilitate the understanding of how entropy values relate to the fetal image regions analysed. We have studied in detail the relationship between all the image regions in the dataset and the entropy values before concluding how to generalise our findings which are reported below.

**Fig. 18:**
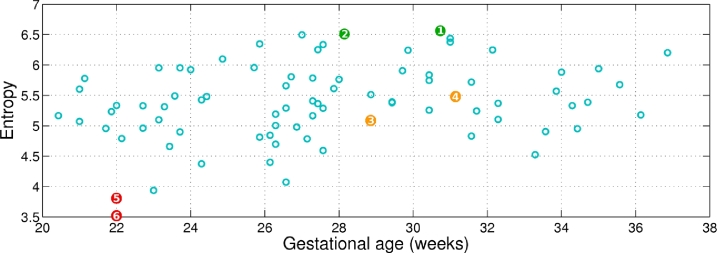
Entropy of the background region across gestational age for all the images in the dataset. The numbers within the coloured bullets correspond to the images in Fig. 19.

**Fig. 19:**
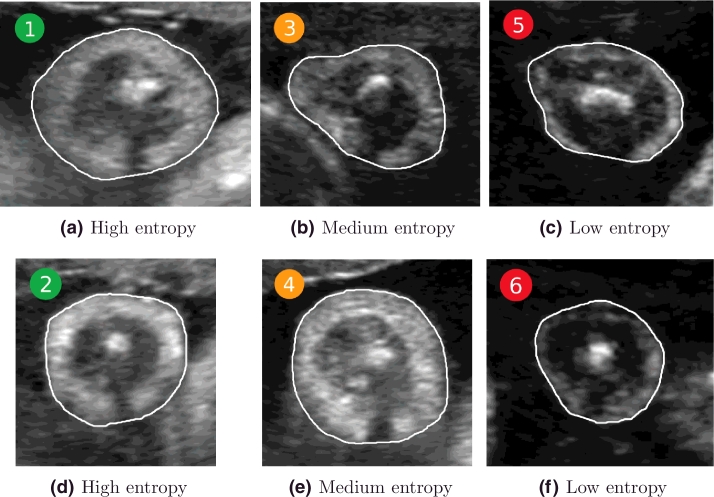
Examples of background entropy as colour-coded in Fig. 18. (a,d) High entropy; (b,e) medium entropy; and (c,f) low entropy. The higher the entropy, the more fetal and maternal tissues surround the adipose tissue layer.

The entropy of the background region is shown in Fig. 18 for all the images in the dataset. A selection of representative images with low, medium, and high background entropy values in Fig. 18 are shown in Fig. 19 to visually appreciate the difference. Observe that higher entropy values are correlated with the presence of more fetal and maternal tissues surrounding the adipose tissue layer. The lower the entropy, the clearer the interface between background and adipose tissue.

**Fig. 20:**
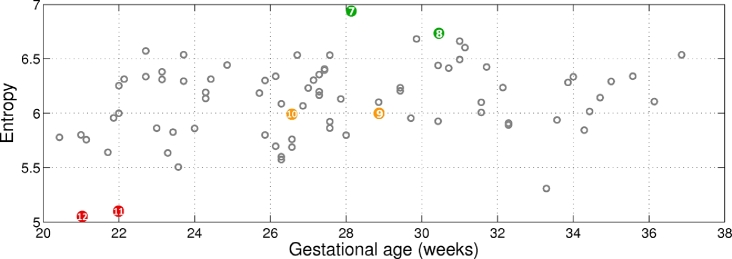
Entropy of the adipose tissue region across gestational age. The numbers within the coloured bullets correspond to the images in Fig. 21. High entropy denotes better image appearance for the adipose tissue region.

**Fig. 21:**
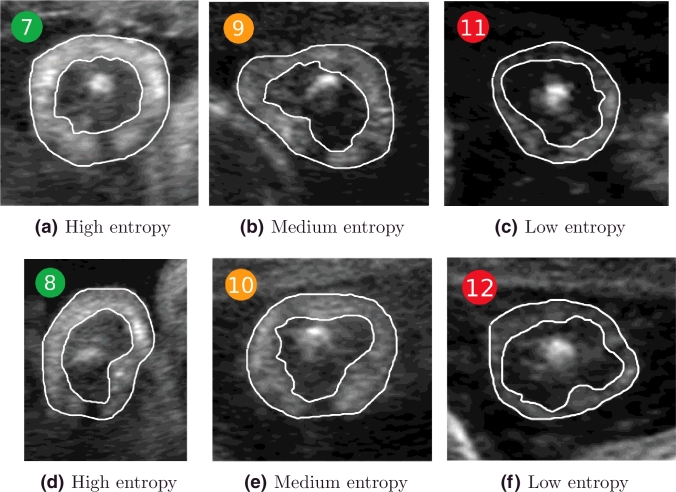
Examples of adipose tissue entropy as colour-coded in Fig. 20. (a,d) High entropy; (b,e) medium entropy; and (c,f) low entropy.

Similarly, the entropy for the adipose tissue region (cf. white region in Fig. 17) across gestational age is shown in Fig. 20, with representative examples for low, medium, and high entropy shown in Fig. 21.

The adipose tissue regions in Fig. 21(a and d) present more information, showing higher intensity levels in these regions. Regions outlined in Fig. 21(c and f) have lower entropy, and their appearance looks fuzzier, visually corresponding to a lower quality. Ideally, we would like the adipose tissue region to be associated with high entropy.

**Fig. 22:**
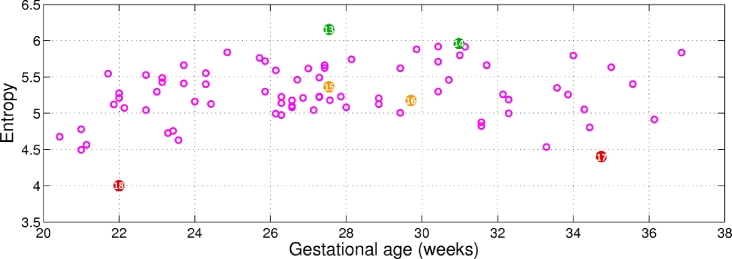
Entropy of the muscle region across gestational age. The numbers within the coloured bullets correspond to the images in Fig. 23. Ideally, we would like the muscle region to have low entropy and a dark appearance.

**Fig. 23:**
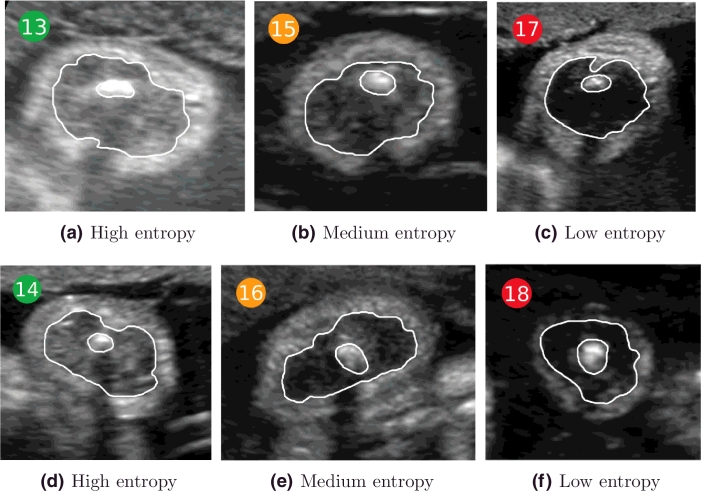
Examples of muscle entropy as colour-coded in Fig. 22. (a, d) High entropy; (b, e) medium entropy; and (c, f) low entropy.

The entropy values for the muscle region (cf. magenta region in Fig. 17) are represented in Fig. 22 across gestation. Examples for low, medium, and high entropy, as indicated in Fig. 22, are shown in Fig. 23. Observe that low entropy muscle regions (Fig. 23(c and f)) are much darker than high entropy muscle regions (Fig. 23(a and d)), showing that the information content in these regions is very different. Ideally, we would like the muscle region to be dark, hence to have low entropy.

**Fig. 24:**
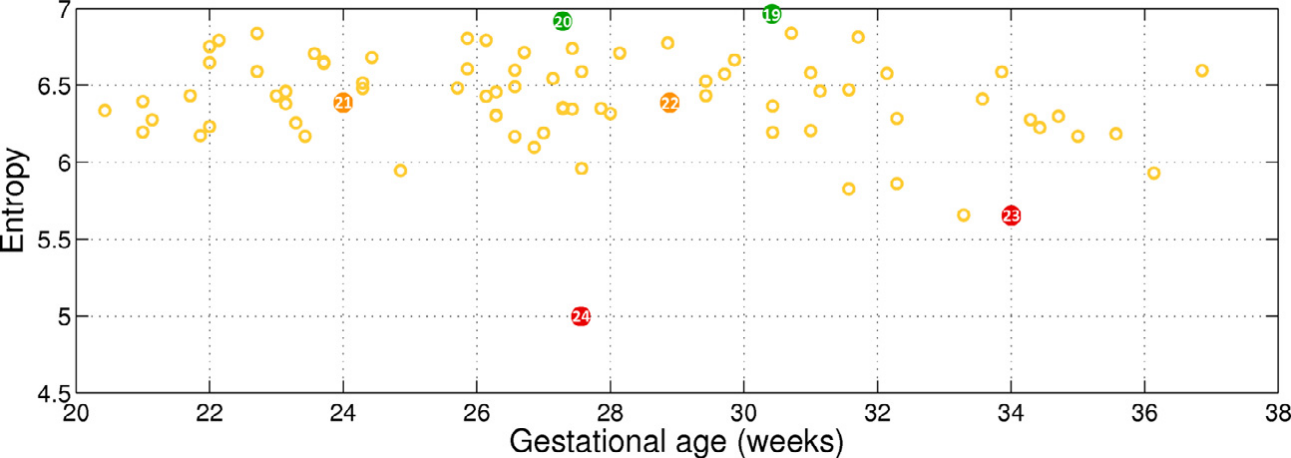
Entropy of the humerus bone region across gestational age. The numbers within the coloured bullets correspond to the images in Fig. 25.

**Fig. 25:**
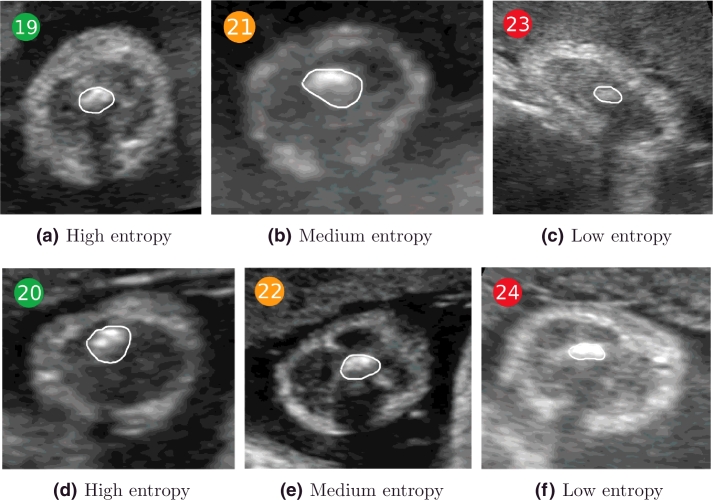
Examples of bone entropy as colour-coded in Fig. 24. (a, d) High entropy; (b, e) medium entropy; and (c, f) low entropy.

The entropy for the humerus bone region (cf. yellow region in Fig. 17) is represented in Fig. 24 across gestation. Representative examples for low, medium, and high entropy, as indicated in Fig. 24, are shown in Fig. 25. In this case, the difference is not as noticeable as for the other regions, due to the small size of the structure. However, it can be seen that regions with lower intensity variability within the region have lower entropy, as shown in Fig. 25(c and f).

Comparing the 4 regions, the highest mean entropy is observed for the humerus bone region, with a value of 6.40 bits. Then, the adipose tissue mean entropy has a value of 6.11 bits, the background region has a mean entropy value of 5.38 bits, and the muscle region has a mean entropy value of 5.27 bits. We conclude from this that, on average, the bone presents the highest information content, followed by the adipose tissue region. Background and muscle regions have lower information content.

**Fig. 26:**
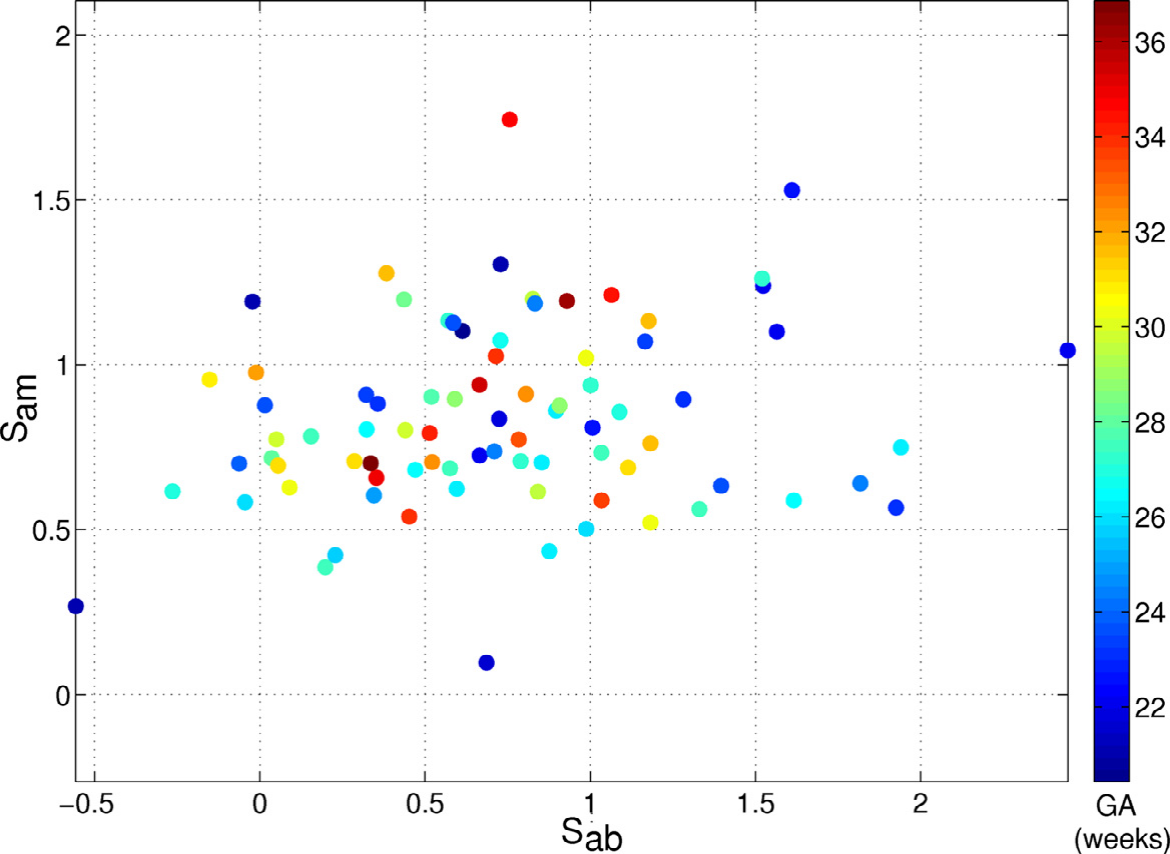
Image quality assessment for the fetal arm dataset. *S*_ab_ denotes the difference of entropy between adipose tissue and background, whereas *S*_am_ represents the difference of entropy between adipose tissue and muscle. Each value has been colour coded with its corresponding gestational age given in weeks. The lower the score values, the more similarity between adjacent regions, and the lower the image quality (bottom left hand side corner of the graph). The higher the score values, the more difference between adjacent regions, and the higher the quality (top right hand side corner of the graph).

Having looked at the entropy (and entropy variation across gestational age) for different tissues of interest we now consider how to define image segmentation quality metrics. Recall, that the goal is to segment the adipose tissue layer. Thus the two interfaces of interest are background–adipose tissue, and adipose tissue–muscle. Therefore, we define two scores to assess the difference of entropy between the background region and the adipose tissue region, and the adipose tissue region and the muscle region for each image in the evaluation dataset. Let *S*_ab_ be the score representing the difference in entropy between adipose tissue and background, defined as

(15) $$\begin{array}{c} S_{\text{ab}}=H\left(A_{\text{adipose}\;\text{tissue}}\right)-H\left(A_{\text{background}}\right); \end{array}$$

and *S*_am_ the score associated with the difference of entropies between adipose tissue and muscle, defined as

(16) $$\begin{array}{c} S_{\text{am}}=H\left(A_{\text{adipose}\;\text{tissue}}\right)-H\left(A_{\text{muscle}}\right), \end{array}$$

with *H*(*A*_adipose tissue_), *H*(*A*_background_), and *H*(*A*_muscle_) as defined in (14). Both scores are useful in assessing the overall image segmentation quality, as shown in Fig. 26, where each value is colour-coded by its corresponding gestational age.

Fig. 26 represents the image quality of each image by using the scores *S*_ab_ and *S*_am_, derived from adjacent regions as previously defined. Score values are low when adjacent regions are similar, and hence image quality overall is lower. Higher score values translate into more distinct adjacent regions, and hence higher image quality overall. Gestational age is incorporated into Fig. 26, as it is normally correlated to image quality (the image quality generally decreases with gestational age). This dataset was chosen to be representative of this particular application, showcasing a variety of image qualities as found in clinical practice. The entropy-based analysis shows that the dataset has a correspondingly high variability in terms of entropy, including several cases with negative *S*_ab_ values, where the entropy in the background is higher than the information of the adipose tissue layer. This can happen when the arm is surrounded by other organs (e.g. limbs, abdomen, placenta) as shown in Fig. 19 for the high entropy examples. These effects are important to consider in the assessment of a segmentation method, as they present challenging conditions for any method.

**Fig. 27:**
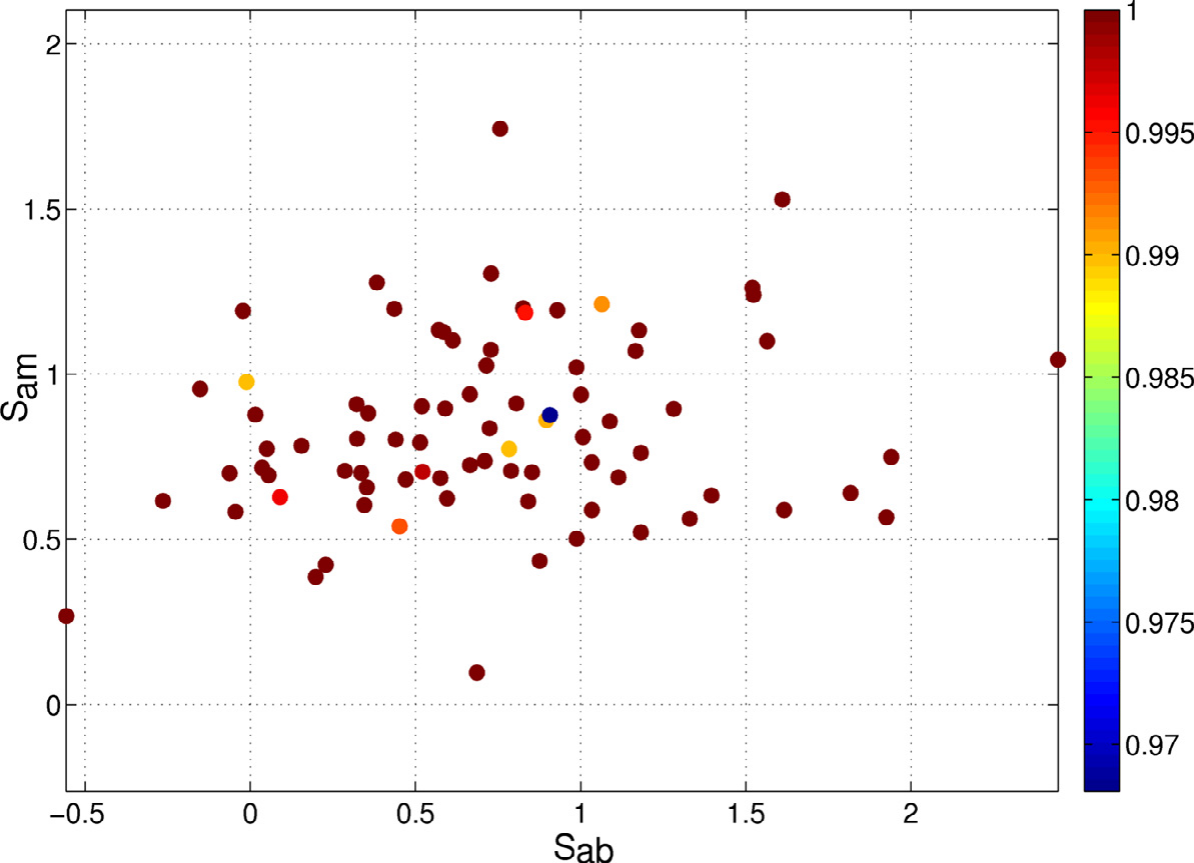
Segmentation precision (repeatability) with respect to image quality scores *S*_ab_ and *S*_am_. Varying the initial seeds result in mostly the same segmented object except for a few cases where small differences appear.

**Fig. 28:**
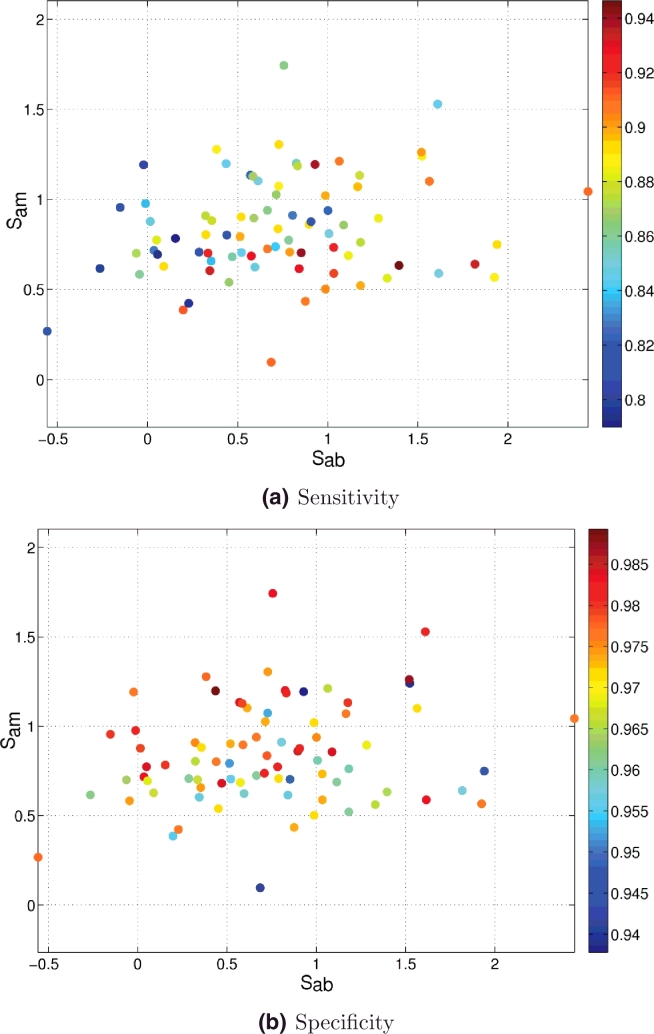
Segmentation accuracy with respect to image quality scores *S*_ab_ and *S*_am_. (a) Sensitivity. (b) Specificity. (For interpretation of the references to colour in this figure legend, the reader is referred to the web version of this article).

Having explained how to define image segmentation quality metrics for our clinical dataset we can now look at how the automated segmentation method performs with respect to these metrics. Figs. 27 and 28 show how precision and accuracy correlate with the two image segmentation metrics, respectively.

The repeatability of the method is presented in Fig. 27. Observe that in most cases the results obtained are very similar when varying the position of the initialisation seeds. Only small differences can be observed in a few cases (values below 1) across the whole range of image qualities.

As shown in Fig. 28 the proposed segmentation method performs robustly over a range of image qualities, giving high values of accuracy in most cases, independently of their image appearance. The lowest accuracy values (blue colours) in terms of sensitivity occurred for images in the bottom left hand side quadrant of Fig. 28.(a), where more similarity between adjacent regions exits (lower quality since adjacent tissue layers look similar) and where the background seems to present more surrounding structures (negative *S*_ab_ values). However, high precision and accuracy values (red and yellow colours) can also be observed in that same quadrant. It is worth pointing out that the lowest accuracy values observed are above 80%, which is good in terms of segmentation performance. Therefore, we conclude that the proposed segmentation method is robust across the variety of image qualities present in the clinical evaluation dataset.

## 4 Discussion and conclusions

This paper has presented three main technical contributions: a feature-based segmentation strategy adapted to US images, a gap completion method, and a novel quantitative image quality assessment approach to assess segmentation performance.

The complete US image segmentation framework introduced in this paper is based on a feature-based fuzzy connectedness segmentation method and requires manual placement of the seeds, after which the remaining steps are performed automatically. The selection of the threshold was fixed for this application, in future might be automated by, for instance, the method of Miranda et al. (2008). The proposed approach uses structural and edge information based on local phase, instead of intensities and intensity gradients, to drive the segmentation. The resulting segmentation is then completed by filling one or more gaps caused by shadows or artefacts in the segmented object of interest using a shape descriptor. A final regularisation based on mean curvature flow is performed to smooth the final contours.

Although more conceptually advanced fuzzy connectedness methods exist, such as RFC and IRFC, it remains to be seen how these would perform in US images. This paper reports results on AFC applied to US images, which is the most basic form of FC with affinities specially formulated for US image segmentation. Once this basic investigation is reported and the behaviour of AFC understood in its most fundamental form, we can then take on investigations to study how more advanced forms of FC with the same forms of affinities on a multi-object setting would perform.

We argued that all segmentation methods should report their results in conjunction with a quantitative image quality analysis to show that the dataset used is representative of a clinical application, and not selected to best suit a particular methodology. A novel quantitative image quality assessment protocol based on entropy was presented and applied to different image partitions to derive interface scores to show the variability of qualities existing in the dataset, representative of a real clinical application. This technique could readily be adapted to suit images from different clinical applications.

A qualitative and quantitative evaluation was performed on 81 cross-sectional images of the fetal arm across gestation, by using region and distance-based metrics. The results showed a similar performance to manual segmentations. Furthermore, the quantitative image quality assessment method showed that the performance of the method was robust across a variety of image qualities representative of a real clinical environment.

The proposed method has undergone clinical assessment on pilot data (Knight, Rueda, Noble, Papageorghiou, 2012, Knight, Rueda, Noble, Papageorghiou, 2012, Rueda, Knight, Papageorghiou, Noble, 2012) and is now part of a large clinical study aimed at establishing normative nutritional growth charts of healthy fetuses across gestation (Knight et al., 2014). The presented framework estimates three main clinical measurements from US images: the amount of fetal arm adipose tissue, the fat-free (lean and bone) areas (useful for body composition assessment), and the adipose tissue percentages for each cross-section (normalised measurements with respect to arm size) across gestational ages. In this study, we have analysed cross-sectional data, but the method is also suitable to study longitudinal data, towards achieving a personalised nutritional monitoring of the fetus.

The 2D feature-based FC implementation could readily be extended to 3D, as local phase and fuzzy connectedness can be easily extended to 3D. Finally, the proposed framework is motivated by, but not limited to this particular application or imaging modality and could equally be applied to other soft tissue segmentation problems, such as myocardium segmentation (Dietenbeck, Alessandrini, Barbosa, D’hooge, Friboulet, Bernard, 2012, Zhu, Papademetris, Sinusas, Duncan, 2010), including contrast-enhanced US (CEUS) images, or intravascular US (IVUS) (Ciompi, Pujol, Gatta, Alberti, Balocco, Carrillo, Mauri-Ferre, Radeva, 2012, Moraes, Furuie, 2011, Zhu, Zhang, Shao, Cheng, Zhang, Bai, 2011).

## Figures and Tables

**Algorithm 1:**
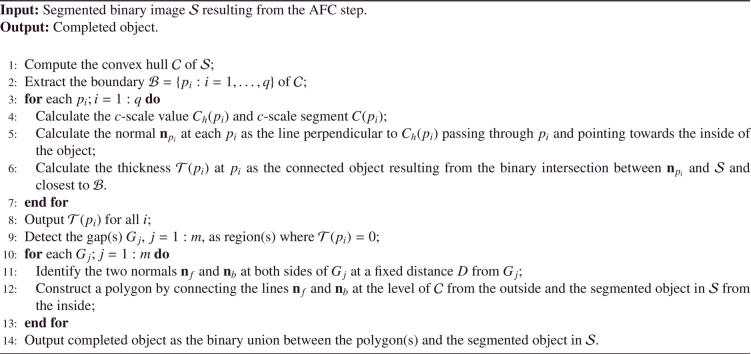
Object completion.

**Table 1:** Intra and inter-observer variability. Manual delineations from Expert 1 (E1) and Expert 2 (E2) are evaluated against themselves and against each other using area overlap and distance-based metrics. The area overlap metrics evaluated are precision, accuracy (sensitivity and specificity), and Dice similarity as defined in Udupa et al. (2006). The distance-based metrics evaluated are the maximum symmetric contour distance (MSD), the average symmetric contour distance (ASD), and the root mean square contour distance (RMSD) as defined in Heimann et al. (2009).

		**Intra-expert**		**Inter-expert**
		**variability**		**variability**
		**E1**	**E2**	**E1 vs E2**
**Precision** (%)		83.49 ± 4.10	87.06 ± 3.06	80.29 ± 3.99
**Accuracy** (%)	Sensitivity	90.15 ± 4.75	94.19 ± 2.72	88.10 ± 5.29
	Specificity	98.11 ± 0.95	98.11 ± 0.93	97.59 ± 1.38
**Dice** (%)		90.95 ± 2.46	93.05 ± 1.77	88.99 ± 2.49
**MSD** (mm)		1.02 ± 0.52	0.93 ± 0.49	1.27 ± 0.68
**ASD** (mm)		0.29 ± 0.13	0.23 ± 0.10	0.36 ± 0.16
**RMSD** (mm)		0.38 ± 0.18	0.31 ± 0.15	0.47 ± 0.22

**Table 2:** Quantitative evaluation. Automatic segmentations (auto) are evaluated against the ground truth, generated from manual delineations from Expert 1 (E1) and Expert 2 (E2), using area overlap and distance metrics. The area overlap metrics evaluated are accuracy (sensitivity and specificity) and Dice similarity as defined in Udupa et al. (2006). The distance-based metrics evaluated are the maximum symmetric contour distance (MSD), the average symmetric contour distance (ASD), and the root mean square contour distance (RMSD) as defined in Heimann et al. (2009).

		**Auto vs E1**	**Auto vs E2**	**Mean**
**Accuracy** (%)	Sensitivity	85.63 ± 4.55	88.98 ± 4.38	87.30 ± 3.84
	Specificity	96.86 ± 1.41	97.23 ± 1.12	97.05 ± 1.17
**Dice** (%)		86.02 ± 2.90	88.21 ± 2.79	87.11 ± 2.60
**MSD** (mm)		1.72 ± 0.86	1.65 ± 0.86	1.68 ± 0.82
**ASD** (mm)		0.46 ± 0.19	0.36 ± 0.18	0.41 ± 0.18
**RMSD** (mm)		0.58 ± 0.24	0.49 ± 0.24	0.54 ± 0.23

## Footnotes

1 http://www.cs.ubc.ca/~mitchell/ToolboxLS/

2 http://www.intergrowth21.org.uk

3 http://www.interbio21.org.uk
